# Compression or expansion of disability among two birth cohorts of US adults with diabetes during the past 20 years?

**DOI:** 10.1016/S2213-8587(16)30090-0

**Published:** 2016-06-10

**Authors:** Barbara H Bardenheier, Ji Lin, Xiaohui Zhuo, Mohammed K Ali, Theodore J Thompson, Yiling J Cheng, Edward W Gregg

**Affiliations:** 1 Division of Diabetes Translation, US Centers for Disease Control and Prevention, Atlanta, GA, USA; 2 Immunization Safety Office, US Centers for Disease Control and Prevention, Atlanta, GA; 3 Merck & Co., North Wales, PA, USA; 4 Hubert Department of Global Health, Rollins School of Public Health, Emory University, Atlanta, GA, USA

## Abstract

**Background:**

The life expectancy of the average American with diabetes has increased, but the level of health and functioning of those extra years are not known.

**Methods:**

Comparing adults aged 50 to 70 with (n=3,027) and without diabetes (n=9,750), we assessed incident disability, remission from disability, and mortality between population-based Cohort 1 (born 1931-1941, followed 1992 to 2002) and Cohort 2 (born 1942-1947, followed 2002 to 2012), from the Health and Retirement Study. Disability was defined by mobility loss, some difficulty with ≥1 instrumental activities of daily living (IADL), and some difficulty with ≥1 activities of daily living (ADL). Age-specific probabilities representing the two birth cohorts in the U.S. were entered into a five-state Markov model to estimate the number of years of disabled and disability-free life by age 70.

**Findings:**

Among persons with diabetes, compared with Cohort 1(n=1,071), Cohort 2 (n=300) experienced more disability-free and total years of life, later onset of disability, and fewer disabled years lost. Solutions to the simulations of the Markov models suggest that among 50 year old diabetic men this amounted to a 0.8 to 2.3 year delay in disability across the 3 metrics (mobility *p*=0.01, IADL *p*=0.24, ADL *p*=0.01), while living 0.7 to 1.3 years longer (mobility p<0.0001, IADL p=0.001, ADL p<0.0001); results were similar for women. Parallel improvements in disabled life were gained across cohorts of non-diabetic adults (cohort 1 n=9,218; cohort 2 n=2,727), although non-diabetic adults in both cohort 1 and cohort 2 had significantly more disability-free years (e.g., cohort 1: non-diabetic men from age 50: 17.0 vs diabetic men: 13.0; cohort 2: non-diabetic men from age 50: 17.9 vs diabetic men: 14.8) and fewer life years lost (e.g., cohort 1: non-diabetic men from age 50: 1.2 vs diabetic men: 2.8; cohort 2: non-diabetic men from age 50: 0.6 vs diabetic men: 1.5) than diabetic adults within the two cohorts (p< 0.0001).

**Interpretation:**

Regardless of diabetes status, adults experienced compression of disability and gains in disability-free life years.

**Funding:**

None

## Introduction

Incidence and prevalence of diabetes have more than doubled in the past two decades in virtually all demographic subgroups of the U.S. population.(1) These trends have been accompanied by large reductions in mortality rates that have increased the number of years spent with diabetes for the average person and for the population overall.(2) As a result, the lifetime probability of diabetes has also increased dramatically, now reaching 40% for both men and women in the United States.(3) Although yearly rates of several diabetes-related complications declined during this period,(4) the impact of living more years of life with diabetes on the quality of those extra years of life remains unclear.

People with diabetes have double the incidence of physical disability, and the few estimates of trends in disability prevalence from surveys among the diabetic population suggest that there has been no change in the past 20 years.(5) Thus, whether recent clinical and public health efforts have been successful in compressing disability (i.e., reducing the number of years with disability) while increasing lifespans remains unclear. Compression occurs if the delay in onset of disability is greater than the increase in life expectancy. As a result, the average time spent in an active state (i.e., disability-free) will increase, both in absolute terms and as a proportion of total life expectancy (TLE).(6) Alternatively, expansion of disability occurs if the gain in TLE is associated with longer periods of disability, implying that medical advances have extended life for those suffering from disabling diseases, without changing the age of disability onset or the number of healthy years of life.

As diabetes is one of the most common conditions associated with increasing rather than decreasing prevalence, we assessed if disability expanded or was compressed among the diabetic population and compared it with that of the non-diabetic population among two consecutive birth cohorts between the ages of 50 and 70 years in the United States. We used a discrete Markov model to determine whether the net impact of recent trends in diabetes incidence, disability, and mortality have affected the average age of disability onset and the number of healthy and disabled years experienced by adults with and without diabetes.

## Methods

We analysed data from the Health and Retirement Study (HRS), a population-based, prospective health interview survey of a cohort of adults ≥ 50 years in the United States.(7, 8) These survey data were collected every two years from 1992 through 2012. We used birth cohorts to assess the net impact of recent trends in diabetes incidence, disability, and mortality. The first cohort (Cohort 1) included 9,750 (n=8,687 without diabetes; n=1,067 with diabetes) respondents born from 1931 through 1941 and surveyed in 1992 (baseline) and biennially through 2002. The second cohort (Cohort 2) included 3,027 (n=2,727 without diabetes; n=300 with diabetes) respondents born from 1942 through 1947 surveyed in 2002 (baseline) and biannually through 2012. Baseline response rates during the study ranged from 70% for cohort 2 to 81% for cohort 1 and the follow-up response rates ranged from 84.0% to 89.1% for cohort 1 and from 87.1 to 90.7% for cohort 2.(9) The HRS is sponsored by the National Institute on Aging and conducted by the Institute for Social Research at the University of Michigan. The Health Sciences Institutional Review Board at the University of Michigan approved the HRS study design.

We developed a simulation model that incorporated the national levels of diabetes prevalence, incidence and mortality among the two birth cohorts. Using the model, we calculated the average age of disability onset, disability-years, and life years lost between age 50 and 70 among the diabetes and non-diabetes populations in the two birth cohorts. To test our hypothesis of no compression or expansion of disability, we compared Cohort 1 (weighted to the 1992 U.S. population of the birth cohort born during 1931-1941, followed through 2002) with Cohort 2 (weighted to the 2002 U.S. population of the birth cohort born during 1942-1947, followed through 2012).

Diabetes was defined as the self-report by a respondent to HRS of a diabetes diagnosis (ie, being told by a doctor that he or she has diabetes or high blood sugar) at first interview or during the study period.(9) Disability was defined using three different disability domains: mobility, instrumental activities of daily living (IADL), and activities of daily living (ADL). Mobility disability was based on responses to questions regarding difficulty: 1) walking one block; 2) walking several blocks; 3) climbing one flight of stairs; 4) stooping, crouching, or kneeling; and 5) pushing or pulling a large object.(10) We defined mobility loss as having difficulty with four or five of these difficulties. In 1992 IADL disability was defined as having difficulty in performing at least one of the following: reading a map, using a calculator, and using a microwave; these are the activities that are assumed to have been used by Wallace and Herzog in their paper.(11) From 1994 through 2012, IADL was defined differently, as difficulty with any of using the telephone, taking medication, handling money, shopping, and preparing meals. Further explanation of how these variables were constructed has been published elsewhere.(7) ADL disability was defined as having difficulty in performing at least one of the following: walking across a room, getting in and out of bed, dressing, bathing, and eating.

Deaths were confirmed using the National Death Index and the Social Security Death Index. To obtain data on the condition of deceased respondents prior to their death, proxies for the respondents were interviewed. Next of kin are interviewed if a respondent dies, and if no next of kin, a friend is contacted. If the estate is not settled at the time of the exit interview, a post-exit interview at the next wave is conducted.

### Statistical analysis

Baseline characteristics were compared cohort 1 with cohort 2, among those with diabetes and among those without diabetes, using chi-squared statistics with 95% confidence intervals and a statistically significant cut-off of *p*<0.05. We conducted logistic regression using generalised estimating equations to obtain annual incidence of disability and mortality according to diabetes status by age group and also to determine the age-specific yearly probability of becoming disabled, dying, and remission to a non-disabled state among those who become disabled. Respondents that entered the study with prevalent disability were excluded from the incidence and remission estimates. We used inverse probability weighting to reduce bias related to missing data, whether it was due to loss of follow-up or non-reporting by the respondent in that wave or previous waves.(12) In this method, logistic regression is used to determine the predicted value of having complete data; weights are the inverse of the probability of having complete data. To obtain these estimates, data were modelled with STATA version 13 (StataCorp, College Station, Texas).

### Markov model with a 20 year time horizon

We developed a discrete-time Markov simulation model with annual transition. The model was applied to two populations, one for those with diabetes and the other for those who remained diabetes-free. The models predict disability-related outcomes from ages 50–70 years, using probability estimates of disability, mortality and remission (figure). During each 1 year interval, individuals may move from and to one of the five states: not disabled, short-term disability, not disabled but with previous disability, long-term disability and death. Because of the high recovery rates observed in the data, we created two bridge states of short-term disability and not being disabled but had disability history. Short-term disability is defined as being disabled less than one year, in contrast with long-term disability defined as remaining disabled more than one year. In the model, individuals with short-term disability revert to not being disabled, die, or continue to be disabled after one year of disability onset. Based upon data from HRS, individuals may have multiple episodes of short-term disability over a lifetime.

To understand the impact of diabetes on twenty-year disability outcomes, we estimated and compared outcomes with those of individuals of the same ages without diabetes that remained without diabetes until death. The confidence intervals of the twenty-year estimates were estimated using Monte Carlo simulation, with 10,000 random draws of age- and sex-specific estimates (5,000 for each sex) from the underlying parametric distributions derived from HRS data.

### Role of the funding source

There was no funding source for this study.

## Results

Cohort 1 included 9,754 respondents, 1,067 of whom had a diabetes diagnosis and 8,687 of whom did not; cohort 2 included 3,027 subjects, 300 of whom had a diabetes diagnosis and 2,727 of whom did not. Age-adjusted diabetes prevalence did not differ between persons born in the 1940's with those born in the 1930's, (9.9% (95% CI, 9.3, 10.4) vs 9.2% (95% CI, 8.1, 10.4). However, BMI, educational attainment, and age each differed by cohort (table 1). Among those with and without diabetes, the baseline prevalence of history of stroke and self-reported high blood pressure at baseline increased significantly from the baseline of the first cohort to the second (*p*<0.05). Among those without diabetes, the prevalence of current smokers declined significantly from 27.2% (95% CI, 25.8, 28.7) to 21.2% (95% CI, 18.8, 23.8).

The proportions of respondents with prevalent ADL and mobility loss at baseline were significantly higher among those with diabetes than among those without. Yet, the prevalence of mobility loss and difficulty with ADLs decreased significantly among those in cohort 2 compared with cohort 1, for those with and without diabetes. IADL differences are not reported at baseline because of the differences in measurements in 1992 from the rest of the interview years.

We found significant differences between cohorts within age group for incidence of disability, recovery from disability, and mortality by disability status, though it varied some by diabetes status (*p*<0.05; table 2). Between the two cohorts, incidence of mobility loss, IADLs and ADLs decreased among those aged 50-60 years and 61-70 years among adults with and without diabetes. Recovery from incident disability increased among both age groups for all disability types, with the exception of mobility loss among those with diabetes aged 50-60 years. Mortality decreased among those with ADLs aged 50-60 years for those with diabetes.

### Healthy and Disabled Life Years

Among diabetic men from ages 50 and 60, there was a significant -1.3 (95%CI: -1.7, -0.8; from 2.8 to 1.5) and -0.3 (95%CI: -0.6, 0.0; from 1.6 to 1.3) year decrease, respectively, in life years lost (i.e., total remaining life years up to age 70) between the two periods, with a delay in the age of onset of disability from age 50 for all disability types and from age 60 for mobility loss and ADLs (table 3 and appendix table). From age 60, diabetic men in Cohort 2 experienced later age of disability onset for all three disability types (0.9 to 1.4 years later in cohort 2), more disability-free years (0.9 to 1.4 years fewer) and fewer disabled years (difference of 0.7-1.1 years) than those in Cohort 1.

Like men with diabetes, non-diabetic men had a postponement of disability onset, along with equivalent increases in healthy years of life and a reduction in years of disabled life from age 60 to 70.

Among diabetic women there was a delay in the age of onset of mobility loss accompanied by more non-disabled years and fewer life years lost. For example, 60 year old diabetic women in Cohort 2 experienced later age of disability onset for all three disability types (1.2 to 1.6 years later), more disability-free years (1.2-1.6 more years) and fewer disabled years (1.1-1.4 fewer) than those in Cohort 1 (table 4). These encouraging findings are similar among non-diabetic women, who also had significant increases in non-disabled years and decreases in disabled years.

All differences between those with diabetes and without diabetes within each cohort by sex, age, and disability type were statistically significantly different (*p*<0.001).

## Discussion

In this comparison of disability and mortality between U.S. adults born in the 1940's versus the 1930's, persons with diabetes are generally becoming disabled later and living more disability-free years by the age of 70. These improvements in health status – suggesting a compression of morbidity – affected adults both with and without diabetes, both sexes, and were observed across different disability definitions. We noted some differences by sex: men gained total years of life from ages 50 and 60, whereas women only had decrease in life years lost from age 50.

The difference in healthy years over time was driven by 3 factors in our model: mortality, incident disability, and remission from disability. With the exception of IADLs, disability-free life years increased among the all groups because mortality risk and disability risk declined in tandem, along with an increase in remission from disability. The non-diabetic population in the first cohort, however, had a greater number of healthy years and later disability onset than their diabetic peers. Even though they had less chance of improvement, those without diabetes in cohort 2 improved significantly compared to cohort 1 in terms of disabled and life years as those with diabetes.

Although our analyses could not assess the factors explaining the compression of disability in the U.S. population, our findings are consistent with the concurrent improvements in the management and control of cardiovascular disease risk factors and HbA1c as well as improved processes of care and reductions in diabetes-related complications and other chronic conditions, such as cardiovascular disease.(13-15) The decreases in mortality of the diabetic population have been previously documented in the United States(2) and in other populations(16), but may also have the effect of suppressing reductions in prevalence of chronic conditions, as was evident in the fact that stroke and heart disease prevalence increased, rather than decreased over time in the diabetic population.

Over the period from 1997 to 2007, diabetes was shown to increasingly contribute to disability among people aged 50 to 64.(17) However, that study assessed the prevalence of disability, unlike our study which excluded prevalent disability cases and assessed incidence of disability and remission from disability which are different metrics. Other reported causes of difficulty with a physical function included depression, heart problems, hypertension, and weight problems. Although our analyses did not assess factors modifying the changes in disability, morbidity, and healthy life years, our analysis of baseline characteristics suggests that increases in BMI, prevalent hypertension, and history of stroke are all plausible factors for the gap between those with diabetes and those without. Class II and class III obesity increased from 73 to 123 percent between the 2 cohorts among diabetic adults while only increasing 31% for both and from a much lower proportion in cohort 1 for those without diabetes; these categories of obesity have previously been shown to carry 1.4 and 1.8 times the prevalence of disability compared to normal weight persons aged over 65 years.(18) Stroke prevalence, which increased 4.7 percentage points in the diabetic population over time, has been associated with a 50 percent increased risk of disability.(19) Although rates of diabetes-related complications have declined substantially over the past two decades, the absolute numbers of diabetic adults with complications persists because of the continued increase in the prevalence of diabetes.(4)

Our findings show that among those without diabetes, more years of total life remaining combined with postponing or recovering from disability results in a greater number of healthy life years. This finding could be related to increases in those following a healthy lifestyle, which prolongs life expectancy and postpones disability.(6) It is also possible, however, that more healthy life years are being achieved due to more healthcare utilisation, and in some cases, expensive procedures. For example, from 1999 to 2008, there was a dramatic increase in the rate of knee replacements (tripling for those 45-64 years and doubling for those ≥ 65 years) in the United States, which substantially reduces pain and improves functioning among people with severe knee problems.(20) Future studies should consider how other aspects of care, whether efficient, low-expense primary care, or advanced therapies for other chronic conditions such as cardiovascular diseases, cancers, and musculoskeletal conditions, are affecting disability rates.

There are several limitations to our study. First, our discrete-time Markov model considered those without diabetes to remain diabetes-free until death. However, the age-specific probabilities used as input into the model included incident cases of diabetes. By using 1-year transition cycle simulations, the age-specific probabilities by diabetes accounted for the incident diabetes cases (i.e., those who did not become disabled prior to diabetes diagnosis, as those who became disabled prior to diabetes diagnosis would have been censored after disability onset). Second, our Markov model does not allow for stratification of the transitions by multiple factors, such as obesity, physical activity, high blood pressure, cardiovascular disease, education, and depression, which could explain many of the differences between those with and without diabetes observed. A separate study of HRS data found lower wealth and education to be associated with higher levels of disability and earlier onset of disability.(21) Thus the greater education attainment achieved in cohort 2 may be associated with the compression of disability and later onset of disability. Third, our analyses were limited by lack of data beyond age 70 in cohort 2, which led us to restrict analyses of both cohorts to the 50 to 70 age-range to permit a valid comparison of the two cohorts. Thus, our analyses should be regarded as examination of fairly early disability, as opposed to more common age-related disability that occurs between age 70 and death. Fourth, data for age at diabetes diagnosis were not available for cohort 2 at baseline. Given that cohort 2 was on average 1 year older than cohort 1 and the prevalence of obesity was much greater among cohort 2, they likely had a longer duration of diabetes which may have affected the onset of disability. Fifth, disability measures were self-reported difficulty with performing tasks which may not be considered ‘disability’ but we used four or five tasks to define severe mobility loss to lessen potential bias. Although IADLs were measured differed during the baseline of cohort one, there was consistency across the remaining the interview years, therefore this difference should pose minimal bias in assessing incidence and remission of IADL difficulty. Finally, our study design does not allow us to separate the effects of period differences from cohort differences, but remains the first study that we are aware of to quantify whether later cohorts with diabetes are experiencing a compression of morbidity relative to earlier cohorts.

Few studies have quantified healthy and disabled life years in adults with diabetes.(22-24) A previous analyses of Health and Retirement Survey data found that persons with diabetes become disabled 6 to 7 years earlier than persons with diabetes. Estimates from the Global Burden of Disease Study (GBD) suggest that diabetes is the 7^th^ leading cause of increased years with disability.(24) However, the GBD estimated disabled life years is based on a combination of prevalence and standard disability weights, which is driven by prevalence and therefore does not lend itself to direct comparison with our estimates, which incorporated individual-level disability incidence estimates and are independent of prevalence. Our analyses are also not directly comparable to studies of trends in disability prevalence because our estimates incorporate incidence of and remission from disability, as well as mortality.(17) We are not aware of previous studies examining trends in healthy and disabled life years among a national sample of these birth cohorts. In addition to having important implications for patients with diabetes and the families and health systems that care for them, trends in healthy years of life serve as an indication of whether clinical and public health goals to compress disability are being achieved. It is unknown whether the compression of disability among those with and without diabetes, will continue. Our analyses only included those born up to 1947; one study examining Baby Boomers (born 1947 and after) found that difficulty with ADLs increased significantly for this generation.(25)

If reduction in mortality occurs because of longer survival with diabetes and disability, then length of life with both will increase. One intervention study among obese adults with type 2 diabetes found that a mean weight loss of 7% or more and increasing duration of physical activity to more than 175 minutes a week led to a reduction of 48% in the severity of mobility-related disability.(10) In addition, a systematic review on interventions to prevent disability in frail community-dwelling adults found that relatively long-lasting and multi-component, several-times weekly physical activity programs for moderately physically frail older persons can be protective against disability.(26) Healthy lifestyle programs including physical activity also reduce the risk of developing clinically significant symptoms of depression and preserve physical quality of life among overweight adults with diabetes.(27) Because of the possible burden of new cases of mobility loss among those with and without diabetes aged 50-64 years, physical activity programs may be helpful in reducing the risk of disability in this age group. In addition, factors leading to disability, such as weight and physical and mental functioning, might be addressed so that the benefits of better health care are optimised. Ultimately, the decrease in disability onset due to primary prevention of diabetes may play an important role in achieving longer disability-free life years.

## Supplementary Material

1

## Figures and Tables

**Figure:**
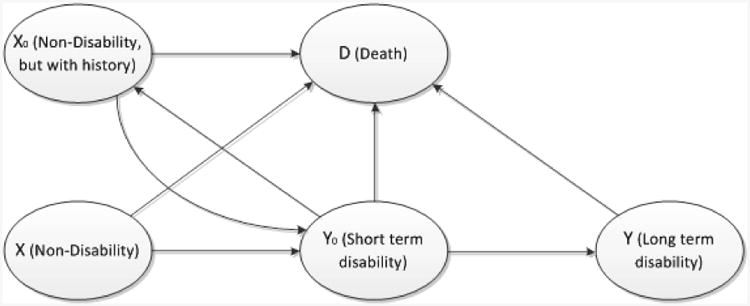
The Markov model for disease progression for a specific sex/diabetes status cohort

**Table 1:** General characteristics of study population according to diabetes status at baseline

	Cohort 1: 1992		Cohort 2: 2002			
	With diabetes % (95% CI) n=1,067	Without diabetes % (95% CI) n=8,687	With diabetes % (95% CI) n=300	*p*-value	Without diabetes % (95% CI) n=2,727	*p*-value
Sex:				0.33		0.78
Male	49.1 (45.5,52.7)	47.5 (46.5, 48.5)	52.9 (45.4,60.2)		47.3 (45.6, 48.9)	
Race / Ethnicity				0.62		0.24
NH White	71.8 (67.2,76.0)	84.4 (82.7, 86.1)	68.6 (61.3,75.0)		83.8 (81.1, 86.3)	
NH Black	18.7 (15.4,22.6)	9.5 (8.6, 10.5)	20.4 (14.4,28.1)		9.0 (7.8, 10.4)	
Hispanics	9.5 (6.9,12.9)	6.1 (4.8, 7.6)	11.0 (7.2,16.6)		7.2 (5.0, 10.1)	
Education				<0.00 01		<0.000 1
< HS	34.9 (31.0, 39.1)	21.7 (20.1, 23.4)	19.9(15.7,25.0)		13.3 (11.5, 15.3)	
HS	48.8 (44.9, 52.6)	55.4 (53.9, 56.8)	53.7 (46.8,60.5)		55.0 (52.4, 57.6)	
> HS	16.3 (14.1, 18.8)	22.9 (21.1, 24.9)	26.4(21.0,32.5)		31.7 (28.9, 34.7)	
BMI				0.03		<0.0001
< 25.0	18.2 (15.8, 21.0)	38.5 (37.2, 39.7)	11.5 (8.6, 15.2)		29.4 (27.0, 32.0)	
25.0 – 29.9	39.6 (37.0, 42.3)	41.3 (40.1, 42.5)	32.3 (27.5,37.4)		40.3 (38.0, 42.7)	
Obesity Class by BMI						
Class I 30.0 – 34.9	25.1 (22.3,28.0)	15.1 (14.2, 16.0)	23.4 (17.7,30.3)		18.9 (17.2, 20.9)	
Class II 35.0 – 39.9	10.7 (8.9, 12.8)	3.5 (3.1, 4.0)	18.5 (14.6,23.2)		7.7 (6.5, 9.0)	
Class III ≥ 40.0	6.4 (5.0, 8.3)	1.6 (1.3, 2.0)	14.3 (10.4,19.4)		3.7 (2.7,4.9)	
Current smoker	24.4 (22.1, 26.8)	27.2 (25.8, 28.7)	22.1 (16.9, 28.2)	0.55	21.4 (19.0, 24.0)	0.14
ADL difficulty	23.0 (19.7, 26.6)	8.4 (7.7, 9.2)	22.3 (17.1, 28.5)	0.83	7.8 (6.8,8.9)	0.28
Mobility loss	24.8 (21.8, 28.1)	9.4 (8.5, 10.4)	20.4 (15.0,27.2)	0.20	4.7 (3.9, 5.8)	<0.0001
Heart disease	21.9 (19.2, 24.9)	8.7 (8.0, 9.4)	28.9 (24.0,34.3)	0.02	10.5 (9.1, 12.0)	0.02
Stroke	5.4 (3.9, 7.3)	1.8 (1.5, 2.2)	14.5 (10.7,19.4)	0.0002	2.9 (2.3, 3.6)	0.0039
High blood pressure	55.5 (51.6, 59.4)	30.0 (28.8, 31.4)	69.5 (62.5,75.7)	0.0011	36.6 (34.2, 39.1)	<0.0001
BMI mean (SD)	30.0 (0.22)	26.7 (0.06)	31.8 (0.46)	0.0014	28.1 (0.16)	<0.0001
Age mean (SD)	56.1 (0.10)	55.5 (0.04)	57.2 (0.12)	<0.0001	57.0 (0.03)	<0.0001

*p*-value: Statistical test of difference between cohort 1 and 2, among those with diabetes and among those without diabetes

Abbreviations: CI: confidence interval; NH: Non-Hispanic; HS: High School; BMI: Body Mass Index; ADL: Activity of daily living; SD: Standard deviation

**Table 2:** Annual incidence of disability and mortality according to diabetes status

	Cohort 1 (born in 1930's)			Cohort 2 (born 1940's)	*p*-value		*p*-value		*p*-value
	50-60 yrs	61-70yrs	Total	50-60 yrs		61-70yrs		Total	
**Diabetes group**									
**Incidence of Disability stratified by age group**									
Mobility loss	6.7 (5.8,7.7)	7.0 (6.2,7.8)	6.7 (6.0,7.4)	4.7 (3.5,5.9)	0.0035	3.5 (2.9,4.1)	<0.0001	3.9 (3.4,4.5)	<0.00 01
IADL	5.7 (4.8,6.6)	6.3 (5.6,7.7)	5.9 (5.3,6.5)	4.3 (3.3,5.3)	0.05	2.9 (2.3,3.6)	<0.0001	3.5 (2.9,4.1)	<0.00 01
ADL	6.8 (5.9,7.7)	7.9 (7.3,8.7)	7.0 (6.3,7.8)	4.4 (3.4,5.3)	0.0005	3.2 (2.6,3.8)	<0.0001	3.7 (3.1,4.2)	<0.00 01
**Incidence of Recovery from Disability stratified by age group**									
Mobility loss	18.4(11.8,24.9)	15.6 (13.1,18.1)	15.9 (13.6,18.2)	23.9 (18.7,29.2)	0.17	23.5 (20.0,26.9)	0.0013	23.5 (20.3,26.8)	0.001 2
IADL	15.3(9.9,20.6)	17.3 (14.4,20.2)	17.3 (14.7,20.1)	26.3 (22.4,30.3)	0.0025	26.7 (22.6,30.8)	0.0001	26.6 (23.7,29.4)	<0.00 01
ADL	12.4 (6.6,18.3)	15.8 (13.5,18.2)	15.7 (13.5,18.0)	24.4 (19.1,29.8)	0.0036	26.1 (23.5,28.7)	<0.0001	25.7 (23.1,28.3)	<0.00 01
**Mortality by incident disability**									
Mobility loss	2.9 (2.0,3.8)	5.8 (4.6,7.1)	4.5 (3.8,5.3)	1.4 (0.2,2.7)	0.07	5.3 (3.6,7.0)	0.55	4.1 (2.9,5.2)	0.25
IADL	3.0 (1.4,4.6)	5.4 (4.3,6.5)	4.4 (3.6,5.2)	1.9 (0.0,4.2)	0.41	4.3 (2.6,6.0)	0.24	3.5 (2.3,4.6)	0.10
ADL	2.4 (1.4,3.4)	5.7 (4.6,6.8)	4.3 (3.6,5.0)	1.1 (0.0,2.5)	0.05	5.3 (3.6,7.0)	0.66	3.9 (2.6,5.3)	0.34
**Non-Diabetes group**									
**Incidence of Disability stratified by age group**									
Mobility loss	2.0(1.7,2.2)	2.6 (2.3,2.8)	2.2 (2.0,2.5)	1.5 (1.3,1.8)	0.01	1.2 (1.0,1.4)	<0.0001	1.4 (1.2,1.5)	<0.00 01
IADL	2.0 (1.8,2.3)	2.6 (2.3,2.8)	2.3 (2.1,2.5)	1.5 (1.2,1.9)	0.0041	1.4 (1.1,1.6)	<0.0001	1.4 (1.2,1.7)	<0.00 01
ADL	2.4 (2.2,2.7)	3.3 (3.1,3.6)	2.8 (2.6,3.0)	1.7 (1.4,2.0)	0.0006	1.5 (1.2,1.8)	<0.0001	1.6 (1.4,1.8)	<0.00 01
**Incidence of Recovery from Disability stratified by age group**									
Mobility loss	18.8(14.2,23.4)	21.0 (23.2,27.0)	20.8 (18.9,22.6)	26.4 (22.0,30.9)	0.03	26.3 (23.8,28.7)	0.0019	26.3 (24.1,28.6)	<0.00 01
IADL	21.8 (16.3,27.3)	21.9 (19.9,23.8)	21.9 (20.0,23.8)	29.4 (27.2,31.6)	0.02	30.9 (28.5,33.3)	<0.0001	30.8 (29.1,32.5)	<0.00 01
ADL	21.5 (17.9,25.1)	23.2 (21.2,25.1)	22.8 (21.1,24.6)	29.7 (26.0,33.5)	0.0047	30.9 (28.4,33.3)	<0.0001	30.6 (28.4,32.8)	<0.00 01
**Mortality by incident disability**									
Mobility loss	1.3 (0.9,1.8)	3.9 (3.2,4.6)	2.9 (2.4,3.4)	1.6 (0.2,3.0)	0.82	3.9 (2.6,5.1)	0.99	3.2 (2.2,4.2)	0.89
IADL	1.1 (0.6,1.7)	3.8 (3.1,4.5)	3.0 (2.4,3.6)	0.4 (0.0,1.1)	0.10	3.8 (2.4,5.1)	0.94	2.6 (2.0,3.5)	0.59
ADL	1.0 (0.7,1.4)	3.1 (2.7,3.6)	2.4 (2.1,2.7)	1.5 (0.3,2.7)	0.46	3.0 (1.8,4.2)	0.84	2.5 (1.7,3.3)	0.84

P-values: compare cohort 1 with cohort 2 within age group and disability metric

Estimates are the annual incidence per 100 person-years, adjusted for sex

ADL: Activities of Daily Living

IADL: Instrumental Activities of Daily Living

**Table 3:** Estimated healthy and disabled years among U.S. men with and without diabetes from baseline age to 70 years

	Cohort 1 1992-2002				Cohort 2 2002-2012							
Baseline Age	Disability- free Years	Disabled Years	Life years lost	Average Age of disability onset	Disability -free Years	*p*- value	Disabled Years	*p*- value	Life years lost	*p*- value	Average Age of disability onset	*p*- value
**Diabetes**												
Mobility loss												
50 years	13.0 (12.3,13.6)	4.2 (3.5,4.9)	2.8 (2.5,3.2)	63.0 (62.3,63.6)	14.8 (13.6,15.7)	0.01	3.6 (2.7,4.8)	0.42	1.5 (1.3,1.9)	<0.0001	64.8 (63.6,65.7)	0.01
60 years	6.8 (6.5,7.0)	1.6 (1.4,1.9)	1.6 (1.4,1.8)	66.8 (66.5,67.0)	7.9 (7.6,8.1)	<0.0001	0.9 (0.7,1.1)	<0.0001	1.3 (1.1,1.5)	0.02	67.9 (67.6,68.1)	<0.0001
IADL Disability												
50 years	13.5 (13.0,14.0)	3.7 (3.1,4.2)	2.8 (2.5,3.2)	63.5 (63.0,64.0)	14.3 (13.0,15.3)	0.24	4.1 (3.1,5.4)	0.45	1.5 (1.3,1.9)	<0.0001	64.3 (63.0,65.3)	0.24
60 years	6.9 (6.7,7.1)	1.6 (1.4,1.7)	1.6 (1.4,1.8)	66.9 (66.7,67.1)	7.8 (7.5,8.1)	<0.0001	0.9 (0.7,1.1)	<0.0001	1.3(1.1,1.5)	0.03	67.8 (67.5,68.1)	<0.0001
ADL Disability												
50 years	12.7 (12.1,13.3)	4.5 (3.9,5.0)	2.8 (2.5,3.2)	62.7 (62.1,63.3)	15.0(13.5,15.9)	<0.0001	3.5 (2.5,5.0)	0.21	1.5 (1.3,1.9)	<0.0001	65.0(63.5,6 5.9)	<0.0001
60 years	6.4 (6.2,6.6)	2.0 (1.8,2.2)	1.6 (1.4, 1.8)	66.4 (66.2,66.6)	7.8 (7.5,8.1)	<0.0001	0.9 (0.7,1.2)	<0.0001	1.3 (1.1,1.5)	0.03	67.8 (67.5,68.1)	<0.0001
**No Diabetes**												
Mobility loss												
50 years	17.0 (16.7,17.3)	1.8 (1.5,2.1)	1.2 (1.1,1.3)	67.0 (66.7,67.3)	17.9 (17.4,18.3)	<0.0001	1.5 (1.1,2.0)	0.22	0.6 (0.5,0.8)	<0.0001	67.9 (67.4,68.3)	<0.0001
60 years	8.6 (8.5,8.7)	0.7 (0.6,0.8)	0.7 (0.6,0.7)	68.6 (68.5,68.7)	9.1 (9.0,9.3)	<0.0001	0.4 (0.3,0.5)	<0.0001	0.5(0.4,0.6)	0.02	69.1 (69.0,69.3)	<0.0001
IADL Disability												
50 years	16.9 (16.6,17.2)	1.9 (1.7,2.2)	1.2 (1.1,1.3)	66.9 (66.6,67.2)	17.4 (16.6,17.8)	0.23	2.0 (1.5,2.8)	0.72	0.6 (0.5,0.8)	<0.0001	67.4 (66.6,67.8)	0.23
60 years	8.5 (8.4,8.6)	0.8 (0.7,0.9)	0.7 (0.6,0.7)	68.5 (68.4,68.6)	9.1 (8.9,9.2)	<0.0001	0.4 (0.4,0.5)	<0.0001	0.5(0.4,0.6)	0.01	69.1 (68.9,69.2)	<0.0001
ADL Disability												
50 years	16.4 (16.1,16.7)	2.4 (2.1,2.7)	1.2 (1.1,1.3)	66.4 (66.1,66.7)	17.6 (17.0,18.0)	<0.0001	1.8 (1.4,2.4)	0.10	0.6(0.5,0.8)	<0.0001	67.6 (67.0,68.0)	<0.0001
60 years	8.3 (8.2,8.4)	1.0 (0.9,1.1)	0.7 (0.6, 0.8)	68.3 (68.2,68.4)	9.0 (8.9,9.1)	<0.0001	0.5 (0.4,0.6)	<0.0001	0.5 (0.4,0.6)	0.01	69.0 (68.9,69.1)	<0.0001

* *p*< 0.05 between the two cohorts within diabetes status and disability type; all differences between diabetes and non-diabetes within sex and disability type were statistically significant *p*<0.001

ADL: Activities of Daily Living

IADL: Instrumental Activities of Daily Living

**Table 4:** Estimated healthy and disabled years among U.S. women with and without diabetes from baseline age to 70 years

	Cohort 1 1992 - 2002				Cohort 2 2002 - 2012							
Baseline Age	Disability- free Years	Disabled Years	Life years lost	Average Age of disability onset	Disability-free Years	*p*- value	Disabled Years	*p*- value	Life years lost	*p*- value	Average Age of disability onset	*p*- value
**Diabetes**												
Mobility loss												
50 years	11.3 (10.5,12.1)	6.8 (6.0,7.6)	1.9 (1.7,2.2)	61.3 (60.5,62.1)	13.2 (11.5,14.5)	0.05	5.7 (4.3,7.4)	0.23	1.1 (0.9,1.5)	<0.001	63.2 (61.5,64.5)	0.05
60 years	6.2 (6.0,6.5)	2.7 (2.5,3.0)	1.1 (0.9,1.2)	66.2 (66.0,66.5)	7.6 (7.4,7.9)	<0.001	1.4 (1.2,1.7)	<0.01	0.9 (0.7,1.1)	0.21	67.6 (67.4,67.9)	<0.001
IADL Disability												
50 years	13.0 (12.4,13.7)	5.1 (4.4,5.8)	1.9 (1.6,2.2)	63.0 (62.4,63.7)	14.1(12.7,15.2)	0.16	4.8 (3.7,6.1)	0.68	1.1 (0.9,1.5)	<0.0001	64.1 (62.7,65.2)	0.16
60 years	6.8 (6.5,7.0)	2.2 (1.9,2.4)	1.1 (0.9,1.2)	66.8 (66.5,67.0)	8.0 (7.7,8.2)	<0.00 01	1.1 (0.9,1.3)	<0.0001	0.9 (0.8,1.1)	0.23	68.0 (67.7,68.2)	<0.0001
ADL Disability												
50 years	12.3 (11.6,13.0)	5.8 (5.1,6.5)	1.9 (1.7,2.2)	62.3 (61.6,63.0)	14.6 (13.1,15.6)	<0.0001	4.3 (3.2,5.8)	0.07	1.1 (0.9,1.5)	<0.0001	64.6 (63.1,65.6)	<0.0001
60 years	6.3 (6.0,6.6)	2.6 (2.4,2.9)	1.1 (0.9,1.2)	66.3 (66.0,66.6)	7.9 (7.6,8.2)	<0.0001	1.2 (1.0,1.4)	<0.0001	0.9 (0.8,1.1)	0.24	67.9 (67.6,68.2)	<0.0001
**No Diabetes**												
Mobility loss												
50 years	16.1 (15.7,16.5)	3.1 (2.7,3.5)	0.8 (0.7,0.9)	66.1 (65.7,66.5)	17.2 (16.2,17.8)	0.04	2.4 (1.8,3.3)	0.15	0.4 (0.3,0.6)	<0.00 01	67.2 (66.2,67.8)	0.04
60 years	8.4 (8.3,8.5)	1.2 (1.1,1.3)	0.4 (0.4,0.5)	68.4 (68.3,68.5)	9.1 (8.9,9.1)	<0.0001	0.6 (0.5,0.7)	<0.0001	0.4 (0.3,0.4)	0.04	69.1(68.9,69.1)	<0.0001
IADL Disability												
50 years	16.6 (16.2,16.9)	2.6 (2.3,3.0)	0.8 (0.7,0.9)	66.6 (66.2,66.9)	17.2 (16.3,17.8)	0.17	2.3 (1.7,3.2)	0.49	0.4 (0.3,0.5)	<0.0001	67.2 (66.3,67.8)	0.17
60 years	8.5 (8.4,8.6)	1.1 (1.0,1.2)	0.4 (0.4,0.5)	68.5 (68.4,68.6)	9.1 (9.0,9.2)	<0.0001	0.5 (0.4,0.6)	<0.0001	0.4 (0.3,0.4)	0.05	69.1 (69.0,69.2)	<0.0001
ADL Disability												
50 years	16.2 (15.8,16.5)	3.1 (2.7,3.4)	0.8 (0.7,0.9)	66.2 (65.8,66.5)	17.4 (16.7,17.8)	<0.0001	2.2 (1.7,2.8)	0.02	0.4 (0.3,0.6)	<0.0001	67.4 (66.7,67.8)	<0.0001
60 years	8.2 (8.1,8.3)	1.3 (1.2,1.5)	0.4 (0.4,0.5)	68.2 (68.1,68.3)	9.1 (8.9,9.2)	<0.0001	0.6 (0.5,0.7)	<0.0001	0.4 (0.3,0.4)	0.05	69.1 (68.9,69.2)	<0.0001

* *p*< 0.05 between the two cohorts by diabetes status and disability type; all differences between diabetes and non-diabetes by sex and disability type were statistically significant *p*<0.001

ADL: Activities of Daily Living

IADL: Instrumental Activities of Daily Living

## Research in context

### Evidence before this study

Mortality rates among people with diabetes are declining, as are yearly rates of several diabetes-related complications, but the impact of living more years of life with diabetes on the quality of those extra years of life remains unclear. Although cross-sectional studies have shown that the disability prevalence among the diabetic population has not changed in the past 20 years, it is unknown if clinical and public health efforts have been successful in compressing disability (ie, reducing the number of years of disability into the last years of life). Few studies have quantified healthy and disabled life years in adults with diabetes. A previous analyses of Health and Retirement Survey data found that persons with diabetes become disabled 6 to 7 years earlier than persons with diabetes.

### Added value of this study

We are not aware of previous studies examining trends in healthy and disabled life years among a national sample of these birth cohorts. In this comparison of disability and mortality between U.S. adults born in the 1940's versus the 1930's, persons with diabetes are generally becoming disabled later and living more disability-free years by the age of 70. These improvements in health status – suggesting a compression of morbidity – affected adults both with and without diabetes, both sexes, and were observed across different disability definitions.

### Implications of all the available evidence

Available evidence related to compression of disability among adults with diabetes suggests that healthy lifestyles that assist in a maintenance of healthy weight and lean to fat ratios are effective in reducing disability among adults with diabetes. Similarly, evidence based approaches to prevent diabetes and cardiovascular disease through both lifestyle modification and preventive care risk factors to reduce CVD are also important for continued reduction in diabetes-related morbidity and disability.
