# Supplementary material for: Compression or expansion of disability among two birth cohorts of US adults with diabetes during the past 20 years?
Source: Lancet Diabetes Endocrinol. Author manuscript; Available in PMC 2017 Aug 1. (PMC4959005; doi:10.1016/S2213-8587(16)30090-0)
Supplement: 1 [file NIHMS796167-supplement-1.docx]

**Table.** Change in healthy and disabled years among U.S. men and women with and without diabetes from baseline age to 70 years.

|  | Men | | | | | | Women | | | | | |
| --- | --- | --- | --- | --- | --- | --- | --- | --- | --- | --- | --- | --- |
|  | Change from cohort 1 to cohort 2 | | | | | | Change from cohort 1 to cohort 2 | | | | | |
| Baseline Age | Disability-free Years | *p-value* | Disabled Years | *p* | Life years lost | *p* | Disability-free Years | *p* | Disabled Years | *p* | Life years lost | *p* |
| **Diabetes** |  |  |  |  |  |  |  |  |  |  |  |  |
| Mobility loss |  |  |  |  |  |  |  |  |  |  |  |  |
| 50 years | 1.8 (0.4,3.0) | <0.01 | -0.6 (-1.7,0.9) | 0.41 | -1.3 (-1.7,-0.8) | <0.01 | 1.9 (-0.1,3.5) | 0.05 | -1.1 (-2.7,0.8) | 0.23 | -0.8 (-1.1,-0.3) | <0.01 |
| 60 years | 1.1 (0.7,1.4) | <0.01 | -0.7 (-1.07,-0.4) | <0.01 | -0.3 (-0.6,0.0) | 0.03 | 1.4 (1.0,1.8) | <0.01 | -1.3 (-1.6,-0.9) | <0.01 | -0.2 (-0.4,0.1) | 0.21 |
| IADL Disability |  |  |  |  |  |  |  |  |  |  |  |  |
| 50 years | 0.8 (-0.6,2.0) | 0.24 | 0.4 (-0.7,1.8) | 0.45 | -1.3 (-1.7,-0.8) | <0.01 | 1.1 (-0.5,2.3) | 0.16 | -0.3 (-1.6,1.3) | 0.68 | -0.8 (-1.1,-0.3) | <0.01 |
| 60 years | 0.9 (0.6,1.3) | <0.01 | -0.7 (-0.9,-0.4) | <0.01 | -0.3 (-0.6,0.0) | 0.03 | 1.2 (0.8,1.6) | <0.01 | -1.1 (-1.4,-0.7) | <0.01 | -0.2 (-0.4,0.1) | 0.23 |
| ADL Disability |  |  |  |  |  |  |  |  |  |  |  |  |
| 50 years | 2.3 (0.7,3.2) | <0.01 | -1.0 (-2.1,0.5) | 0.21 | -1.3 (-1.7,-0.8) | <0.01 | 2.3 (0.7,3.5) | <0.01 | -1.5 (-2.8,0.1) | 0.07 | -0.8 (-1.1,-0.3) | <0.01 |
| 60 years | 1.4 (1.0,1.7) | <0.01 | -1.1 (-1.4,-0.8) | <0.01 | -0.3 (-0.6,0.0) | 0.03 | 1.6 (1.2,1.9) | <0.01 | -1.4 (-1.8,-1.1) | <0.01 | -0.2 (-0.4,0.1) | 0.24 |
| **No Diabetes** |  |  |  |  |  |  |  |  |  |  |  |  |
| Mobility loss |  |  |  |  |  |  |  |  |  |  |  |  |
| 50 years | 0.9 (0.3,1.4) | <0.01 | -0.3 (-0.8,0.2) | 0.22 | -0.6 (-0.8,-0.4) | <0.01 | 1.1 (0.0,1.8) | 0.04 | -0.7 (-1.5,0.3) | 0.15 | -0.4 (-0.5,-0.2) | <0.01 |
| 60 years | 0.5 (0.3,0.7) | <0.01 | -0.3 (-0.4,-0.2) | <0.01 | -0.2 (-0.3,-0.1) | 0.02 | 0.7 (0.5,0.8) | <0.01 | -0.6 (-0.7,-0.4) | <0.01 | 0.0 (-0.2,0.0) | 0.04 |
| IADL Disability |  |  |  |  |  |  |  |  |  |  |  |  |
| 50 years | 0.5 (-0.3,1.1) | 0.23 | 0.1 (-0.5,0.9) | 0.72 | -0.6 (-0.8,-0.4) | <0.01 | 0.6 (-0.3,1.4) | 0.17 | -0.3 (-1.0,0.6) | 0.49 | -0.4 (-0.5,-0.2) | <0.01 |
| 60 years | 0.6 (0.4,0.7) | <0.01 | -0.4 (-0.5,-0.2) | <0.01 | -0.2 (-0.3,-0.1) | 0.01 | 0.6 (0.5,0.8) | <0.01 | -0.6 (-0.7,-0.4) | <0.01 | 0.0 (-0.2,0.0) | 0.05 |
| ADL Disability |  |  |  |  |  |  |  |  |  |  |  |  |
| 50 years | 1.2 (0.5,1.7) | <0.01 | -0.6 (-1.1,0.1) | 0.10 | -0.6 (-0.8,-0.4) | <0.01 | 1.2 (0.5,1.8) | <0.01 | -0.9 (-1.5,-0.2) | 0.02 | -0.4 (-0.5,-0.2) | <0.01 |
| 60 years | 0.7 (0.6,0.9) | <0.01 | -0.5 (-0.7,-0.4) | <0.01 | -0.2 (-0.3,-0.1) | 0.01 | 0.9 (0.7,1.0) | <0.01 | -0.7 (-0.9,-0.6) | <0.01 | 0.0 (-0.2,0.0) | 0.05 |

p-values between the two cohorts within diabetes status and disability type;

ADL: Activities of Daily Living

IADL: Instrumental Activities of Daily Living
